# Academy of Stomatology

**Published:** 1905-10

**Authors:** 

**Affiliations:** Academy of Stomatology


### ACADEMY OF STOMATOLOGY.

A special meeting was held Tuesday, June 20, 1905, at eight P.M., the President, Dr. D. N. McQuillen, in the chair.

### INCIDENTS OF PRACTICE.

*Dr. Dudley Guilford.*—I have some bur-holders that I procured in Paris that may be of interest. They are gotten up by Raymenfrayer. The holder consists of a nickel-plated brass frame, into which are put the glass tubes with the head of the bur sticking up. It is a neat arrangement, and at the bottom there is a sterilizing apparatus. I have here a drawer with the same arrangement, except that the tubes are placed in a little frame in the drawer.

Another arrangement which I have not seen before is that of keeping the Swiss broaches wrapped in cotton in a Petrie plate, by which means they can be sterilized dry.

Another useful device is a cotton-holder, which I also procured in France, though it may be an American invention.

*Dr. Otto E. Inglis.*—The remarks of Dr. Guilford suggest a little point which I make use of. It is sometimes necessary to sterilize the broach quickly, and this is rather better accomplished by placing the broach in water and then dipping it into dry sodium. This gives a more reasonable assurance that the cotton is sterile.

*Dr. Boenning.*—I think the suggestion of Dr. Inglis is a good one. I believe that one reason why we have unsatisfactory results is that we have not been sufficiently careful to sterilize instruments and materials. Thorough sterilization is almost as necessary as in abdominal surgery.

I would like to speak of a case of a child who at four years of age, and though in perfect health, has lost her lower centrals. She is now four and a half years of age, and is losing the four molars. There is no history of any injury, and the child is well nourished. I have assured the mother that there is no doubt about the second teeth coming in. I have seen probably one or two cases in which the deciduous teeth were lost at five years, but not in a child of four years. The upper teeth are loosening, and will probably be lost within the next six months. The roots of the teeth lost were absorbed. Dr. Pearce, whom I consulted, has seen two or three cases, and he assures me that the permanent teeth came through normally.

*Dr. Otto E. Inglis.*—Dr. Boenning's case recalls one of a child six years of age with a very deformed mouth and no occlusion at all. There is some hypertrophy of the alveolar process, and the teeth are in such bad condition that I have decided to have chloroform administered and extract all of the deciduous teeth. I had another case of a lady who had a reasonable number of teeth, which looked fairly well, but there was no occlusion whatever. The occlusion was between the ramus of the lower jaw and the tuberosity of the superior maxilla. I have been unable to secure an impression of her mouth.

*Dr. Boenning.*—I have received a letter asking for my experience with the apparatus for drawing pus from the tooth socket. The writer of the letter had used it on a patient and had drawn what appeared to be pus. He was much elated, but the next day the patient returned, saying that he had suffered greatly through the night. Dr. Gaylord, I understand, has been making some experiments with the apparatus, and I would like to inquire of his success.

*Dr. Gaylord.*—I purchased one of the appliances made by the Keystone Electric Company similar to the one shown by Dr. McPherson, and used it in a most pronounced case in which the pus had burrowed through the bone. The mucous membrane in certain lights had a yellowish cast, which would prove to any one that pus was present. With the electrode applied over this area there was gathered an accumulation of material which resembled pus. It was about of the consistency of pus, was of a greenish-yellow color, and I think anybody would have called it pus. A specimen was submitted to a bacteriologist and pathologist whose opinion no one would question. He made a careful examination, and said that no pus was present. The only abnormality was its richness in albumin; otherwise it would have been called saliva. There was nothing to indicate that it was pus. The fact bears out my argument, the night the subject was presented, that things are not always what they appear and that it is better to go at things carefully and with scientific investigation prove them rather than to guess at the results. I have the apparatus, and would be willing to sell it at a greatly reduced price.

*Dr. Otto E. Inglis.*—I have seen and used the apparatus once, and do not desire to purchase Dr. Gaylord's. The gentleman on whom I used the apparatus had the tooth opened in the usual way, but without relief. Suction was made and an effort made to get through the apical foramen. For twenty minutes I applied this battery which had been left at my office by the Keystone Company. No relief was secured, and the tooth was subsequently removed. I could see nothing having the appearance of pus. What I did see was a little frothing about the gum. Personally, I cannot see why the electricity would not follow the easiest channel, going through the soft parts and creating electrolysis.

*The President.*—Personally I have had no experience. Dr. Head tells me that he is very well satisfied with his results in its use.

*Dr. Gaylord.*—I think the instrument is of value as a counter-irritant.

*The President.*—One patient told me that the pain from its use was worse than the pain he was bearing.

*Dr. Inglis.*—My patient complained of a great deal of burning from the use of the apparatus.

Otto E. Inglis,
*Editor Academy of Stomatology.*

